# Catching wandering minds with tapping fingers: neural and behavioral insights into task-unrelated cognition

**DOI:** 10.1093/cercor/bhab494

**Published:** 2022-01-17

**Authors:** Josephine M Groot, Gábor Csifcsák, Sven Wientjes, Birte U Forstmann, Matthias Mittner

**Affiliations:** Department of Psychology, UiT – The Arctic University of Norway, Tromsø 9037 , Norway; Integrative Model-Based Cognitive Neuroscience Research Unit, University of Amsterdam, Amsterdam 1018 WB , The Netherlands; Department of Psychology, UiT – The Arctic University of Norway, Tromsø 9037 , Norway; Department of Experimental Psychology, University of Ghent, Ghent 9000 , Belgium; Integrative Model-Based Cognitive Neuroscience Research Unit, University of Amsterdam, Amsterdam 1018 WB , The Netherlands; Department of Psychology, UiT – The Arctic University of Norway, Tromsø 9037 , Norway

**Keywords:** approximate entropy, executive function, fMRI, mind wandering, pupillometry

## Abstract

When the human mind wanders, it engages in episodes during which attention is focused on self-generated thoughts rather than on external task demands. Although the sustained attention to response task is commonly used to examine relationships between mind wandering and executive functions, limited executive resources are required for optimal task performance. In the current study, we aimed to investigate the relationship between mind wandering and executive functions more closely by employing a recently developed finger-tapping task to monitor fluctuations in attention and executive control through task performance and periodical experience sampling during concurrent functional magnetic resonance imaging (fMRI) and pupillometry. Our results show that mind wandering was preceded by increases in finger-tapping variability, which was correlated with activity in dorsal and ventral attention networks. The entropy of random finger-tapping sequences was related to activity in frontoparietal regions associated with executive control, demonstrating the suitability of this paradigm for studying executive functioning. The neural correlates of behavioral performance, pupillary dynamics, and self-reported attentional state diverged, thus indicating a dissociation between direct and indirect markers of mind wandering. Together, the investigation of these relationships at both the behavioral and neural level provided novel insights into the identification of underlying mechanisms of mind wandering.

## Introduction

The phenomenon of mind wandering in humans can be described as the spontaneous stream of consciousness that comprises thoughts, emotions, and memories (Smallwood and Schooler 2015) that are often related to personal goals and concerns (Shepard 2019) and pervasively occur during daily life and experimental tasks (Killingsworth and Gilbert 2010; Seli, Beaty, et al. 2018). Unsurprisingly given this broad definition, mind wandering has been studied in a wide range of settings and under different labels, for example, stimulus-independent, spontaneous, and task-unrelated thought (Callard et al. 2013). Researchers have identified important dimensions of mind wandering, including intentionality (Seli et al. 2016), emotional valence (Banks et al. 2016), temporality (Maillet et al. 2017), and meta-awareness (Schooler et al. 2011). Here, we define mind wandering as self-generated thoughts that arise independently from external sensory input and pertain to any content that is unrelated to the task athand.

Notwithstanding the diversity of contexts in which mind wandering has been previously investigated, researchers continue in their pursuit to further unravel its underlying neural mechanisms and its effect on other cognitive processes and behavior. In particular, although there is little debate regarding the involvement of executive functions in mind wandering in general, there is no consensus on exactly how mind wandering interacts with executive control systems and whether it is better characterized as executive function use (Teasdale et al. 1995; Smallwood and Schooler 2006) or as the result of executive failure (McVay and Kane 2010; Kane and McVay 2012). Whereas some behavioral research has associated mind wandering with failures of executive control processes (Smallwood et al. 2004; McVay and Kane 2009), converging evidence from neuroimaging studies suggest that mind wandering recruits widespread cortical networks involved in goal-directed behavior, including the frontoparietal control network (FPCN) and dorsal and ventral attention networks (Christoff et al. 2009; Fox et al. 2015; Dixon et al. 2018; Turnbull et al. 2019). Interestingly, there are even reports of associations between task-related attention, as opposed to mind wandering, and greater activation of the default mode network (DMN; Esterman et al. 2014; Kucyi et al. 2017; Groot, Boayue, et al. 2021), a network previously considered to mainly engage during resting-state and self-referential processing (Raichle 2015). Furthermore, a recent resting-state fMRI study with experience sampling demonstrated that activity in the DMN was associated with self-generated thoughts that were not independent from the external environment whereas activity in the dorsal attention network (DAN) related specifically to increases in perceived control over spontaneous thought (Van Calster et al. 2017). Together, these findings undermine the assumption that task-related and task-unrelated states of mind can be independently partitioned into specific functional networks and warrant the development of sensitive behavioral paradigms that disentangle the complex interplay between executive functions and forms of spontaneous thought.

The majority of mind wandering research reports data from self-reports through periodical thought probing and performance errors, usually during a sustained attention to response Task (SART; Christoff et al. 2009; Christoff 2012; Hawkins et al. 2019; Boayue et al. 2019). However, the SART is often slow paced and target stimuli are presented infrequently, preventing more fine-grained tracking of ongoing fluctuations in both attention and executive control. Arguably, a more suitable paradigm to investigate executive functioning is the random number generation task (RNGT; Baddeley et al. 1998) as it is assumed that the generation of random sequences of numbers or letters requires highly controlled executive processes that strategically monitor and inhibit habitual tendencies in order to avoid repetition of response patterns (Jahanshahi et al. 2000; Joppich et al. 2004; Jahanshahi et al. 2006; Peters et al. 2007). Indeed, competing processes such as mind wandering or dual task performance result in the reduced ability to produce such random behavior (Teasdale et al. 1995; Boayue et al. 2020). Thus, investigation of the relationships between the degree of sequence randomness and the occurrence of mind wandering episodes has the potential to provide insights into how the mental processes supporting departures from a task-focused state compete with the cognitive resources needed for executive task performance.

Besides monitoring executive function use, findings from several studies suggest trial-to-trial response time variability as a promising and sensitive marker for fluctuations in attentional focus (Bastian and Sackur 2013; Jubera-Garcia et al. 2019; Zanesco et al. 2020), especially when combined with a monotone and simplistic finger-tapping task that facilitates mind wandering (Seli et al. 2013; Kucyi et al. 2017). Building on these findings, Boayue et al. (2020) recently developed a fast-paced paradigm that combines both these aspects of behavior into a finger-tapping random-sequence generation task (FT-RSGT), allowing ongoing assessment of the degree of self-generated randomness as well as behavioral variability at high temporal resolution. In a series of experiments, it was demonstrated that both measures were consistently related to mind wandering in opposite ways: variability in finger-tapping increased whereas sequence randomness decreased prior to self-reports of mind wandering throughout thetask.

Similarly, several studies have attempted to identify psychophysiological markers reliably related to an individual’s attentional state. In particular, a growing body of evidence suggests that spontaneous changes in pupil size are linked to dynamic fluctuations between internal versus external attention and awareness (Laeng et al. 2012; Schneider et al. 2016; DiNuzzo et al. 2019). Changes in slowly fluctuating baseline pupil size as well as fast evoked pupillary responses are thought to be modulated by the locus coeruleus–norepinephrinergic system (LC/NE; Aston-Jones and Cohen 2005; Joshi et al. 2016) and have introduced new opportunities to objectively monitor mind wandering and arousal state (Mittner et al. 2014; Unsworth and Robison 2016). However, research on the relationship between mind wandering and tonic pupil size has yielded more inconsistent results as both larger and smaller tonic pupils have been associated with mind wandering (Smallwood et al. 2012; Grandchamp et al. 2014; Konishi et al. 2017; Jubera-Garcia et al. 2019). This is possibly due to differences in task demands and thereby the required levels of vigilance (Unsworth and Robison 2018) or, as proposed in a recent theoretical model, variations in tonic pupil size may reflect qualitatively distinct task-unrelated states (Mittner et al. 2016).

In summary, the DMN, attention and executive control networks, and the LC/NE-system are all implicated in mind wandering but empirical evidence into how these neural systems interact to give rise to mind wandering is at present incomplete. Building on previous findings, we aimed to address this by employing an fMRI version of the FT-RSGT that combines experience sampling with objectively defined measures interpreted as indirect makers for changes in ongoing attentional state, including sensitive behavioral indices and pupillometric measures. Following Boayue et al. (2020), we expected increases in the variability of finger-tapping and decreases in the degree of randomness of the tapping-sequence preceding self-reported mind wandering episodes. Furthermore, we aimed to validate that performance of the FT-RSGT indeed relies on executive control processes that are known to be recruited during the original RNGT. To this end, we contrasted brain activation during the generation of random tapping-sequences with a simple alternating finger-tapping task and expected to observe greater activation in frontoparietal regions involved in executive control during random finger-tapping.

Additionally, we explored the pattern of neural activation in relation to both direct and indirect markers of mind wandering by directly assessing the patterns of network-wide activity in the periods preceding experience sampling probes as well as the brain regions that correlated with behavioral performance. In line with a previous finger-tapping study, we expected to observe recruitment of ventral and dorsal attention networks and cerebellum when variability in finger-tapping was high and DMN activation when finger-tapping was more stabilized (Kucyi et al. 2017). The degree of sequence randomness was expected to correlate with activity in frontoparietal and sensorimotor areas associated with executive control and self-determined action (Schubert et al. 1998; Jahanshahi et al. 2000). Direct predictions regarding network activation preceding self-reports of mind wandering are less evident considering the contradictory findings in the literature (Christoff et al. 2009; Mittner et al. 2014; Groot, Boayue, et al. 2021), but similar patterns of neural recruitment are expected for direct (self-report) and indirect (objective task performance and pupil dynamics) measures of mind wandering given that these relationships are replicated on the behavioral level. Finally, dynamic changes in tonic and phasic pupil size were assessed and related to self-reported mind wandering throughout the task. Whereas phasic pupil responses to task-related events are generally expected to be smaller during mind wandering due to perceptual decoupling (Smallwood et al. 2011; Unsworth and Robison 2018), the exact relationship with tonic pupil size is less clear. We therefore also investigated the neural substrates of both pupillary components to explore whether the brain regions correlating with changes in tonic and phasic pupil size would demonstrate similarity to the pattern of neural activity associated with mind wandering.

## Materials and Methods

### Participants

The study was approved by the Ethics Review Board of the Faculty of Social and Behavioral Sciences at the University of Amsterdam. Participants were 27 healthy adult volunteers aged 20–45 years (15 male, mean age = 27.5, *SD* = 7.2 years) who were recruited from the Amsterdam ultra-high field adult lifespan database (AHEAD; Alkemade et al. 2020). Participants had normal or corrected-to-normal vision, no self-reported (history of) psychiatric or neurological illness, and no contraindications for MRI as assessed with a standard safety questionnaire. To avoid biases in task performance related to individual differences in rhythmic abilities and finger tapping, experienced and (semi-)professional musicians were excluded from the study. Written informed consent was obtained prior to the experiment and participation was compensated with a standard monetary reward of €15 for a total duration of 90 min. All materials, anonymized data, and code are publicly available in an Open Science Framework (OSF) repository (Groot, Csifcsák, et al. 2021).

### Finger-Tapping Random-Sequence Generation Task

**Figure 1:**
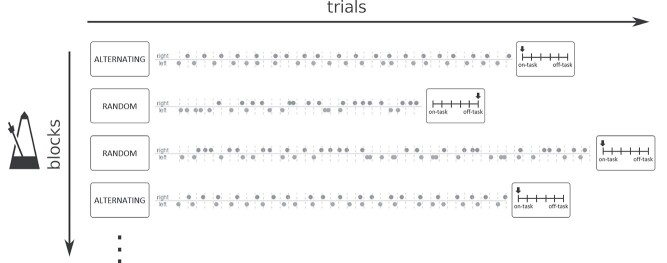
Schematic of the finger-tapping random-sequence generation task (FT-RSGT). Participants produced either alternating sequences of left versus right finger-tapping (alternating blocks) or generated sequences trying to maximize their randomness (random blocks). The rhythm of finger-tapping was continuously indicated by an auditory stimulus paced at 750 ms (metronome). At the end of each block, participants were probed to report to what degree their thoughts were unrelated to the task on a six-point Likert scale (reproduced from Groot, Csifcsák, et al. 2021).

Participants completed 18 experimental and 9 control blocks of the finger-tapping random-sequence generation task (FT-RSGT, Boayue et al. 2020; Fig. 1) in a pseudorandomized order. Stimuli were presented on a 32 inch BOLD screen using PsychoPy (Peirce 2007). At the start of each block, instructions appeared for 4000 ms at the center of the screen to indicate whether it was an experimental (“RANDOM”) or control (“ALTERNATING”) block. In the alternating task, participants were instructed to simply press the response buttons with their left and right index fingers in an alternating sequence (L-R-L-R-L-R-etc.). In the random task, they were asked to generate a sequence of left and right button presses with maximum unpredictability, or randomness (e.g., L-R-L-L-L-R-etc.). Hence, the two tasks were identical with respect to stimulus presentation and execution of motor responses but differed in the randomness criterion and thus, in the extent to which executive control processes were necessary to maintain performance. The concept of randomness was explained with a coin flip analogy: Similar to flipping a coin, a left versus right button press should occur at equal probability and be independent from past or future button presses. To ensure that participants understood these instructions, they performed a short practice run of the task and answered quiz-questions about the concept of randomness (e.g., “If there have been three right presses, must there always be a left press?”). If mistakes were made, further instructions and practice were provided until the task was mastered.

Throughout the experiment, participants had to synchronize their finger-tapping with an ongoing metronome that was presented as an auditory stimulus (440 Hz pitch for 75 ms) every 750 ms. Previous experiments determined that this pace is optimal for engaging in generating random sequences compared to slower and faster metronomes (Boayue et al. 2020). During finger-tapping, participants fixated on a centered fixation cross that was presented on a gray background while attending to the stimuli through MR-compatible headphones. Every block consisted of 80 stimuli on average (range = 74–87) and ended with a thought probe so that the onsets of thought probes were pseudorandomized to occur between 55.5 and 65.3 s (60 s on average). Thought probes were presented for 6000 ms plus a random jitter between 0 and 1000 ms, formulated as: “Where was your attention (i.e., your thoughts) focused just before this question?”. Responses to the thought probes were ordered on a six-point Likert scale with the following annotations: “clearly on-task,” “partly on-task,” “slightly on-task,” “slightly off-task,” “partly off-task,” and “clearly off-task”. To indicate their answer, participants pressed left and right response buttons to navigate an arrow pointing at the categories. The starting point of the arrow on either extreme end of the Likert scale was randomized across thought probes. Participants were explicitly instructed that “off-task” included all thoughts unrelated to the task, for example, daydreaming, personal memories, or future plans whereas “on-task” referred to task-related thoughts, such as thinking about which button to press next or focusing the rhythm of the metronome. The total duration of the task was ~30 min.

### Acquisition and Preprocessing

#### Behavior

Two aspects of FT-RSGT performance were assessed. First, behavioral variability (BV) was calculated as the standard deviation of the intertap intervals (ITIs) of the 25 finger-taps preceding each thought probe. No filtering or preprocessing was performed so that missing or double taps per trial were included in the calculation. The raw standard deviations were log-transformed to approximate a normal distribution. Second, the degree of randomness in the self-generated sequence of left and right finger-taps was measured with the approximate entropy (AE) metric (Pincus and Kalman 1997). As the generated sequences during alternating blocks were completely predictable (i.e., AE = 0), AE was calculated only for the random task condition. Specifically, AE quantifies the regularity in a sequence by evaluating the conditional probability that subsequences of length *m* that are similar remain similar for subsequences augmented by one position (more details on the calculation of AE are in Supplementary Material A). A previous study showed that AE (*m* = 2) measured in the FT-RSGT correlated with the entropy measure in a keyboard version of the original RNGT, validating its use as an index for executive control (Boayue et al. 2020). AE was calculated for every thought probe across the same preceding 25-tap window and transformed as −ln(ln2 − AE). Both BV and AE were then standardized (*Z*-scored) across subjects (i.e., the grand mean and standard deviations were used for standardization). The choice of the 25-tap window was decided a priori based on the assumption that mind wandering occurs in slowly fluctuating episodes spanning multiple seconds as well as for ensuring that sufficient data was gathered for reliable calculations of BV and AE. Additionally, previous experiments revealed that BV and AE based on this window size had the strongest relationship with self-reported mind wandering compared to shorter windows (Boayue et al. 2020).

#### Pupillometry

The pupil area of the left eye was concurrently recorded during the fMRI session at a sampling rate of 1000 Hz with the Eyelink 1000 Plus tracking system (SR Research). The pypillometry package (Mittner 2020) was used to determine subject-specific velocity profiles for blink detection based on the algorithms described by Mathôt (2013). Additional parameters were fine-tuned based on visual inspection of individual datasets, including the margin around blink onset and offset for linear interpolation and maximum distance in time between consecutive blinks for merging. Data were then filtered with a zero-phase shift Butterworth low-pass filter at 5 Hz that was set at 3 Hz for 12 datasets and at 2 Hz for three datasets as visual inspection revealed the presence of abundant high-frequency noise in the pupil signal. These steps ensure rigorous and optimized preprocessing of the pupil signal, circumventing artifacts associated with the high inter-individual variability in blinking transients and frequency. However, due to excessive blinking or technical issues with pupil tracking, the quality of six datasets remained inadequate and these subjects were therefore excluded from all further pupillometric analyses.

Selecting specific windows for extracting the mean pupil signal or peak amplitude is complicated in fast-paced task designs due to the build-up of evoked pupil responses that resemble increases in baseline pupil size and therefore contaminate the baseline estimates of subsequent trials. Therefore, to produce more valid estimates of single-trial tonic (baseline) and phasic (evoked) pupillary dynamics, a recently developed deconvolution-based approach was applied (Mittner 2020). First, the data were downsampled to 250 Hz. Tonic fluctuations were estimated using B-spline basis functions constrained to pass through high prominence troughs in the pupil signal. A second iteration of this estimation, following subtraction of the first tonic estimate as well as modeled pupil–response functions (PRF; Hoeks and Levelt 1993) located at known task events, ensured that the final tonic estimate constituted a smooth curve that remained below the signal on which the phasic pupil responses are superimposed. Single-trial tonic pupil size was then calculated at every stimulus onset. To model phasic pupil responses to task-related events, regressors for every stimulus and tap onset were convolved with the pupil-response function (PRF; *h* = *t^n^e*^*-n*/*t*max^, where *n* = 10 and *t*_max_ = 900; Hoeks and Levelt 1993) and fitted with a nonnegative least-squares solver (Lawson and Hanson 1987) to recover the amplitude of phasic responses as estimated *b* coefficients. However, predictor multicollinearity was observed as stimulus and tap onsets occurred close in time. Therefore, the final single-trial phasic pupil responses were calculated as the sum of *b* coefficients from all events located within the 200-ms window before and after each stimulus onset. Finally, single-trial tonic and phasic pupil responses were standardized (*Z*-scored) within subjects to remove incidental differences in absolute pupil size across subjects. More details on the deconvolution-based pupil analysis are described by Mittner (2021), and an implementation is provided in the pupillometry package (Mittner 2020).

#### Functional Neuroimaging

Participants were scanned with a 3Tesla Philips Achieva MRI system with a 32-channel head coil. *T*_1_-weighted (*T*_1_w) images were acquired with a turbo field-echo (TFE) sequence in 257 sagittal slices (FOV = 256 × 240 × 180 mm [F-H × A-P × R-L], TR = 11 ms, TE = 5.1 ms, acquired voxel size = 0.7 × 0.76 × 0.7 mm, reconstructed voxel size = 0.67 × 0.67 × 0.7 mm). Whole-brain functional images were acquired in a single fMRI run with single-shot fast field-echo (FFE) echo-planar imaging (EPI), collecting 56 transverse slices per volume with 0.2 mm slice gap (FOV = 224 × 224 × 123 mm, TR = 1800 ms, TE = 30 ms, flip angle = 70°, voxel size = 2 mm isotropic). An additional EPI field map with opposite phase-encoding direction was acquired to measure and correct for field distortions.

Imaging data were preprocessed with fMRIPrep v1.1.7 (Esteban et al. 2018) using Nipype v1.1.3 (Gorgolewski et al. 2011). Structural (*T*_1_w) images were corrected for intensity non-uniformity with N4BiasFieldCorrection (ANTs v2.2.0; Tustison et al. 2010), skull-stripped with antsBrainExtraction using the OASIS target template, and spatially normalized to the ICBM 152 Nonlinear Asymmetrical template version 2009c (MNI152Nlin2009cAsym; Fonov et al. 2009) using the nonlinear registration tool in antsRegistration (Avants et al. 2008). Brain tissue was segmented in cerebrospinal fluid (CSF), white matter (WM), and gray matter (GM) using FAST (FSL v5.0.9; Zhang et al. 2001). The functional images were corrected for susceptibility distortion with 3dQwarp (AFNI; Cox and Hyde 1997), using a deformation field estimated from the two EPI references with opposing phase-encoding directions. The unwarped BOLD reference based on the estimated susceptibility distortion was then co-registered to the T_1_w reference with FLIRT (FSL v5.0.9; Jenkinson and Smith 2001) using the boundary-based registration cost-function (Greve and Fischl 2009) and 9 degrees of freedom to account for remaining BOLD distortions. Head-motion parameters (rotation and translation) were estimated with MCFLIRT (FSL v5.0.9; Jenkinson et al. 2002). The preprocessed data were resampled back to native space as well as to standard space (MNI152Nlin2009cAsym template) and smoothed with a 6 mm full-width half-maximum Gaussian kernel using SUSAN (Smith and Brady 1997). All subsequent fMRI analyses were performed on the smoothed preprocessed timeseries in standard space.

### Data Analysis

#### Bayesian Hierarchical Ordered Probit Regression Models

The relationships between self-reported mind wandering, behavioral performance, and pupillary dynamics were assessed with regression models using the thought probe responses from the experimental blocks (random task) of the FT-RSGT as dependent variable. Treating the ordinal probe responses as continuous introduces a range of statistical problems, including poor model fitting, low power, increasing the risk for type I and II errors, and spurious interaction effects (Liddell and Kruschke 2018). To circumvent these issues, we applied Bayesian hierarchical ordered probit regression (Boayue et al. 2019, 2020; Bürkner and Vuorre 2019; Alexandersen et al. 2021) using the brms package (Bayesian Regression Models using Stan; Bürkner 2017). This method models the probability of each discrete point rather than relying on the assumption that the probe responses are normally distributed. In addition, within-subject variability in mind wandering can be taken into account with subject-level random intercepts. As a consequence, probit models are more suitable and sensitive to detect effects in Likert-scale data. For each regression coefficient, we report the posterior mean, its 95% highest-density interval (HDI), and the evidence ratio in favor of a positive (ER_+_) or negative (ER_−_) effect. The ER is calculated as the ratio between the probability of the effect being positive divided by the inverse probability of the effect being zero or negative (ER_+_) or the inverse of that ratio (ER_−_) and can be interpreted as an odds-ratio. We consider an effect as reliable when the area under the marginal posterior distribution that is larger than zero (for ER_+_) or smaller than zero (for ER_−_) is >0.95 (corresponding to an ER of 19 and the 95% HDI excluding zero). The models were fitted with four Hamiltonian Monte Carlo (HMC) chains, each with 1000 warm-up and 4000 post warm-up samples.

In the first probit model, the effects of time (probe number), BV, AE, and the BV × AE interaction on 486 thought probe responses (27 subjects × 18 blocks) were modeled. In accordance with our hypotheses, for time, BV, and BV × AE, we evaluated the evidence for the effect to be larger than zero (ER_+_) and for AE to be smaller than zero (ER_−_). Similarly, the effects of tonic and phasic pupillary dynamics as well as the tonic × phasic interaction were assessed in addition to time in a second regression model using the 18 random blocks from the 21 subjects with complete pupil datasets. For every thought probe, the extracted single-trial tonic and phasic features were averaged across the preceding 25 trials (18.75 s), ignoring trials with more than 40% missing pupil data. This criterion resulted in exclusion of six thought probes, therefore the model was fitted on 372 thought probe responses in total. The evidence for effects larger than zero (ER_+_) for tonic pupil size and the tonic phasic interaction and smaller than zero (ER_−_) for phasic pupil responses was evaluated.

#### fMRI Analysis: General Linear Models

Whole-brain general linear models (GLM) were fitted to the fMRIPrep-preprocessed time series using FSL FEAT (Woolrich et al. 2001) to explore differences in brain activity between the two task conditions and to investigate the role of brain regions involved in mind wandering using 1) experience sampling probes, 2) task performance, and 3) pupillary dynamics. All first-level GLMs included four task-related regressors (left and right finger-taps, metronome stimuli, and thought probe onsets) that were convolved with a double-gamma hemodynamic response function (HRF). In addition, fMRIPrep-derived nuisance regressors calculated for every volume were added, including mean time courses in CSF and WM masks, framewise displacement (FD), six rotation and translation parameters, and discrete-cosine transform (DCT) basis functions to model low-frequency scanner drifts. The modeled data were obtained via ordinary least-squares linear regression. Second-level analyses were performed with FLAME (Beckmann et al. 2003) to obtain group-level parametric contrast maps. Statistical significance of brain areas was evaluated with cluster z-thresholding (Friston et al. 1994). First, a primary voxel-level threshold of z > 2.3 defined clusters of above-threshold voxel activations. Second, a cluster-level threshold of *P* < 0.05 was applied to eliminate non-significant clusters.

First, we explored the hypothesis that random-sequence generation recruits more widespread executive and attentional networks compared to alternating finger-tapping. In addition to the task-related and nuisance regressors, the occurrence of random and alternating blocks was modeled by two boxcar functions convolved with a double-gamma HRF. In a second model, patterns of brain activity associated with episodes of mind wandering compared to on-task thoughts were investigated by convolving boxcar functions that modeled the 10s intervals preceding off-task versus on-task thought probes (Christoff et al. 2009). To account for individual differences in response tendencies, the six probe response categories were dichotomized using an algorithm that determined subject-specific boundaries, where the split-point for each subject was chosen to set the proportion of off-task versus on-task probes as close to 50% as possible. This approach allowed us to identify potential episodes of mind wandering in subjects that selected only a very narrow range of response categories to reflect their current attentional state, possibly due to some degree of satisficing, primacy, and social desirability biases (Weinstein 2018; Weinstein et al. 2018). For example, if a subject exclusively answered with “clearly on-task” and “partly on-task,” responses in the latter category were labeled as off-task in the analysis. The total proportion of off-task reports was 49% using the split-point algorithm whereas this proportion was 36% when collapsing the first three categories into on-task and the other three into off-task. With the latter approach no significant brain activations preceding off-task reports could be observed. Since the two task conditions were presumed to differ in terms of executive resources necessary for performance and may therefore interact differently with mind wandering, probe regressor functions were created separately for random and alternating blocks.

**Figure 2:**
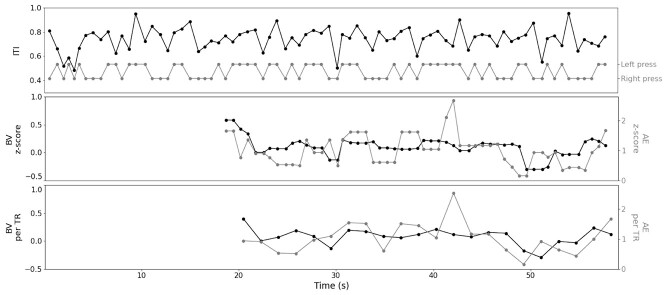
Behavioral performance during a random task block from a single subject (80 stimuli), showing inter-tap intervals (ITI) and left versus right button presses (*top*), used to calculate behavioral variability (BV) and approximate entropy (AE) starting at the 25th stimulus per block (*middle*), which were then linearly interpolated and resampled to the resolution of the fMRI timeseries for the GLM analysis (*bottom*) (reproduced from Groot, Csifcsák, et al. 2021).

Third, regressors for task performance were modeled to evaluate brain activity corresponding to increases and decreases in BV and AE. Starting at the 25th trial (metronome onset) per block, BV and AE (random blocks only) were calculated for every single trial based on the preceding 25 finger-taps. To create parametric regressors, single-trial BV and AE were transformed and standardized across subjects before nearest-neighbor interpolation and resampling to the resolution of the fMRI timeseries (Fig. 2). The use of 25-tap sliding windows resulted in smooth and time-lagged regressor functions that did not require additional convolution with a canonical HRF. Our window size of 25 taps was determined a priori based on previous studies on the latency of mind wandering and on-task episodes (Bastian and Sackur 2013; Pelagatti et al. 2020), in order to ensure comparable measures for BV and AE to the behavioral analysis, and considering the slow nature of the physiological BOLD response. In the final GLM, we explored changes in brain activity associated with increases and decreases in tonic and phasic pupillary dynamics. The single-trial tonic and phasic pupil features were interpolated with nearest-neighbor interpolation and resampled to the resolution of the fMRI timeseries (TR). Whereas tonic pupil was already modeled as a smooth and slowly fluctuating signal, phasic responses were convolved with a double-gammaHRF.

For every resulting group-level statistical map we calculated the overlap with the 7- network parcellation (Yeo et al. 2011), the Harvard-Oxford subcortical structural atlas (Harvard Center for Morphometric Analysis), and the probabilistic cerebellar atlas (Diedrichsen et al. 2009) in standard MNI152 space. The cortical network parcellation included visual (VIS), somatomotor (SOM), dorsal attention (DAN), salience/ventral attention (VAN), limbic (LIM), control (CON), and default mode (DMN) networks. The subcortical parcellation consisted of thalamic nuclei, striatum, pallidum, hippocampus, amygdala, nucleus accumbens, and brainstem and were combined into a general subcortical mask for calculating the total percentage overlap and illustration purposes. All three atlases were resampled to the resolution of the functional timeseries using nearest-neighbor interpolation and binarized. The percentage of voxels in the statistical contrast maps that overlapped with each of the binarized atlases was then calculated, ignoring above-threshold voxel clusters that were located in cerebral white matter. Therefore, regardless of the size of significant clusters, every contrast map was always fully accounted for by the parcellation.

## Results

### Effects of Task Condition on Mind Wandering Reports and Performance

In total, 36% of probe responses were in one of the three off-task categories. The mean probe response given by participants was 2.89 (*SD* = 1.46, median = 3) on the six-point Likert-scale, demonstrating that participants reported that their thoughts were more often focused on performing the task rather than being engaged in mind wandering. Indeed, five subjects never reported that their thoughts were in any of the three off-task categories. There was no significant difference in mind wandering propensity between the random (*M* = 2.86, *SD* = 0.95) and alternating task conditions (*M* = 2.93, *SD* = 1.14, *t*(26) = −0.53, *P* = 0.602). Mean behavioral variability was, however, significantly higher during random (*M* = 0.11, *SD* = 0.57) compared to alternating finger-tapping (*M* = −0.07, *SD* = 0.53, *t*(26) = 3.30, *P* < 0.01) suggesting that the additional task of generating random sequences interfered with maintaining synchronized motor responses to the externally cued rhythm.

### Task Performance and Pupil Dynamics Relate to Mind Wandering Reports

The unadjusted Bayesian *R*^2^ for the first probit model was 0.56 [0.52, 0.59]. In line with expectations, the coefficients for time (*b* = 0.09 [0.07, 0.11], ER_+_ = $\infty$), BV (*b* = 0.33 [0.21, 0.45], ER_+_ = $\infty$), and BV × AE (*b* = 0.07 [−0.05, 0.17], ER_+_ = 7.18) were positive whereas the coefficient for AE (*b* = −0.09 [−0.20, 0.02], ER_−_ = 18.61) was negative. Thus, mind wandering self-reports were more frequent as the task progressed and were preceded by increases in tapping variability and decreases in self-generated sequence randomness although the HDI of the latter coefficient did not exclude zero. The direction of the BV × AE interaction suggests that the relationship between BV and mind wandering was stronger at high levels of AE and weaker (but still positive) at low levels of AE. However, the HDI of this effect included zero, warranting a cautious interpretation of this result. Since BV was significantly higher in the random task, we assessed whether task condition modulated the relationship between BV and mind wandering. We performed a probit model with a BV × task interaction (*b* = −0.04 [−0.21, 0.14], ER_−_ = 1.98), which indicated that the observed positive relationship between BV and mind wandering was independent from task condition.

For the probit model including pupil regressors, the unadjusted Bayesian *R*^2^ was 0.53 [0.47, 0.57]. The positive effect of time on mind wandering was replicated (*b* = 0.08 [0.06, 0.11], ER_+_ = $\infty$). Although phasic pupil responses related to mind wandering in the expected direction (*b* = −0.13 [−0.39, 0.13], ER_−_ = 5.39), tonic pupil size decreased as self-reports of mind wandering increased (*b* = −0.12 [−0.28, 0.04], ER_+_ = 0.08). However, both effects can be considered inconclusive as the HDIs did not exclude zero. Instead, the tonic × phasic interaction (*b* = −0.36 [−0.65, −0.07], ER_+_ = 159) provided evidence of a negative interaction effect. This finding suggests that the relationship between phasic responses and mind wandering is dependent on fluctuations in tonic pupil size. Specifically, the negative relationship between phasic pupil responses and mind wandering, as would be expected due to perceptual coupling, only exists when tonic pupil size ishigh.

**Figure 3:**
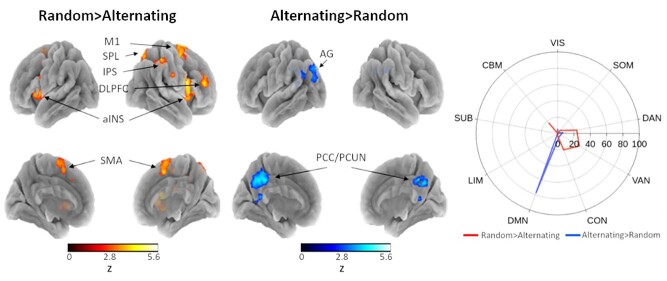
Activations during random-sequence generation contrasted with alternating finger-tapping (*left*) and vice versa (*middle*) and percentage of contrast maps that overlap with a 7-network cortical, subcortical, and cerebellar parcellation (*right*). M1 = primary motor cortex; SPL = superior parietal lobe; IPS = intraparietal sulcus; DLPFC = dorsolateral prefrontal cortex; aINS = anterior insula; SMA = supplementary motor area; AG = angular gyrus; PCC/PCUN = posterior cingulate cortex/precuneus; SUB = subcortex; CBM = cerebellum (reproduced from Groot, Csifcsák, et al. 2021).

### Random Sequence Generation Recruits Executive and Attentional Networks

When contrasted with random-sequence generation, we observed that alternating finger-tapping was associated with localized activity in the posterior cingulate cortex/precuneus and left angular gyrus, both regions that are core nodes of the DMN. In line with our hypothesis, the generation of random tapping sequences instead revealed widespread recruitment of cortical areas generally attributed to attention and executive control networks, including the superior parietal lobe, intraparietal sulcus, primary motor cortex, and dorsolateral prefrontal cortex of the right hemisphere as well as bilateral anterior insula, bilateral medial supplementary motor areas, and bilateral anterior and posterior parts of the cerebellum (Fig. 3, Supplementary Material B). Together, these results provide evidence that the two tasks are qualitatively different regarding the cognitive resources necessary for performance and that random-sequence generation during the FT-RSGT requires the recruitment of brain regions associated with executive functioning.

### Mind Wandering Signatures Diverge for Direct versus Indirect Markers

**Figure 4:**
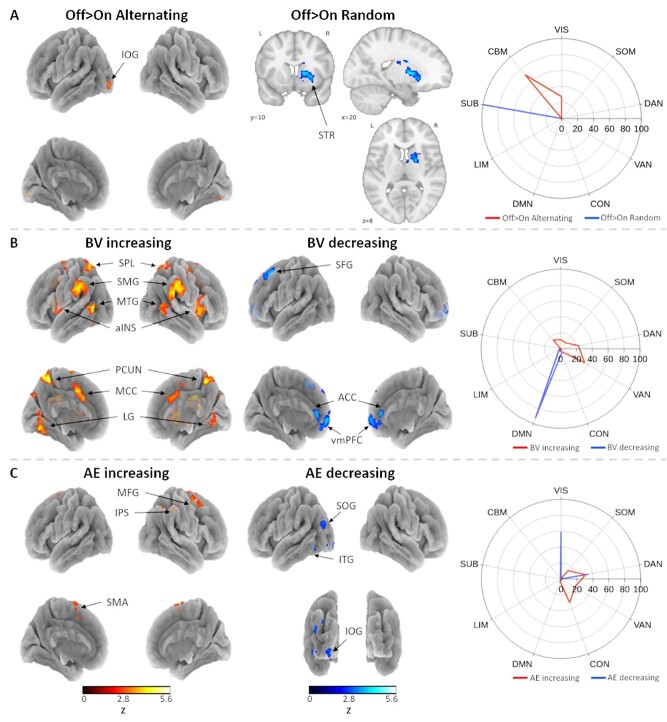
(*A*) Active brain regions preceding mind wandering reports during the alternating (*left*) and random (*middle*) task conditions. (*B*) Regions correlating with increases (*left*) and decreases (*middle*) in behavioral variability. (*C*) Regions correlating with increases (*left*) and decreases (*middle*) in approximate entropy. (*right*) Percentage of contrast maps that overlap with a 7-network cortical, subcortical, and cerebellar parcellation. IOG = inferior occipital gyrus; STR = striatum; SPL = superior parietal lobe; SMG = supramarginal gyrus; MTG = middle temporal gyrus; aINS = anterior insula; SFG = superior frontal gyrus; ACC = anterior cingulate cortex; vmPFC = ventromedial prefrontal cortex; MFG = middle frontal gyrus; IPS = intraparietal sulcus; SOG = superior occipital gyrus; ITG = inferior temporal gyrus; SMA = supplementary motor area; SUB = subcortex; CBM = cerebellum (reproduced from Groot, Csifcsák, et al. 2021).

Contrary to previous findings, brain activations directly preceding self-reports of mind wandering when contrasted with on-task reports could not be localized to the known cortical nodes of either the DMN or FPCN/DAN. Instead, we observed clusters of brain activation associated with mind wandering in visual, cerebellar, and subcortical areas that could be distinguished between the two task conditions. Specifically, mind wandering in the alternating task was preceded by activity in the left inferior occipital gyrus, temporal subgyral white matter (not plotted on the surface mesh), and parts of the bilateral anterior and posterior cerebellum, whereas mind wandering during the random task was associated with greater activity in the right striatum (Fig. 4*A*, Supplementary Material B). To test whether these results were influenced by the selection of the selected data window, the same analysis was performed using 18 s preprobe intervals which resulted in similar activation patterns. In addition, the analysis was repeated combining both task conditions to assess whether separation of the tasks influenced the observed neural correlates of mind wandering. Across tasks, we observed significant activation mainly in the right striatum preceding mind wandering reports when contrasted to on-task reports (Supplementary Material C), showing high overlap (Dice similarity coefficient = 0.68) with the activations preceding mind wandering in the random task condition.

Next, we assessed patterns of neural activity corresponding to variability in finger-tapping (Fig. 4*B*, Supplementary Material B) and obtained strikingly similar results as reported in a previous rhythmic finger-tapping study (Kucyi et al. 2017). Increases in finger-tapping variability were correlated with activity in dorsal and ventral attention networks, including the superior parietal lobes, supramarginal gyri, posterior middle temporal gyri, anterior insula, midcingulate cortices, precuneus, lingual gyri of both hemispheres, and bilateral anterior cerebellum. Furthermore, less variable finger-tapping and thus more optimized task performance was associated with greater activity in left superior frontal gyrus, bilateral anterior cingulate cortex, and bilateral ventromedial prefrontal cortex, thereby mostly mapping to theDMN.

Similarly, we observed correlated neural activity in the expected frontoparietal regions for increases in the degree of randomness in the tapping-sequence as quantified with AE, namely within the right intraparietal sulcus, right posterior middle frontal gyrus, and left medial supplementary motor area, thus showing a similar pattern of neural network recruitment as observed for the random task condition in general. Decreases in AE, signaling decrements in task performance, were instead associated with the left inferior temporal sulcus (poorly visible on the plotted surface mesh) and the superior and inferior occipital gyri of the left hemisphere (Fig. 4*C*). Similar results were obtained when the analyses were performed using 20-tap and 10-tap sliding windows for BV and AE regressor calculation, indicating that the observed patterns of neural activation are robust against changes in this analysis parameter.

### Pupillary Dynamics Map to Subcortical and Visual Cortical Areas

**Figure 5:**
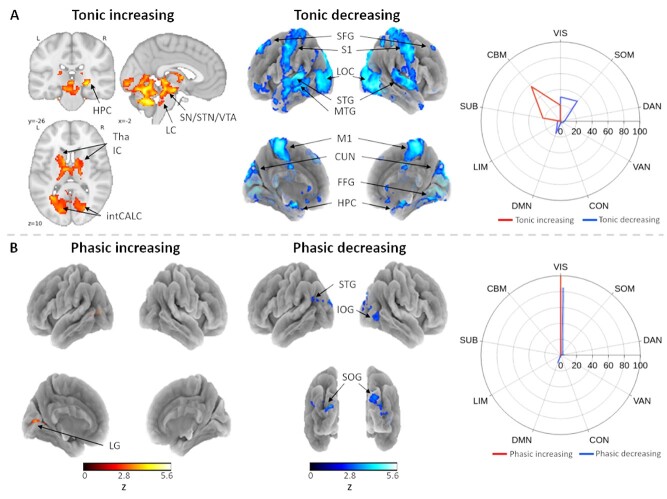
(*A*) Active brain regions with increasing (*left*) and decreasing (*middle*) tonic pupil size. (*B*) Regions correlating with increasing (*left*) and decreasing (*middle*) phasic pupil responses to task-related events. (*Right*) Percentage of contrast maps that overlap with a 7-network cortical, subcortical, and cerebellar parcellation. HPC = hippocampus; LC = locus coeruleus; SN = substantia nigra; STN = subthalamic nucleus; VTA = ventral tegmental area; Tha = thalamus; IC = internal capsule; intCALC = intracalcarine cortex; SFG = superior frontal gyrus; S1 = primary somatosensory cortex; LOC = lateral occipital cortex; STG = superior temporal gyrus; MTG = middle temporal gyrus; M1 = primary motor cortex; CUN = cuneal cortex; FFG = fusiform gyrus; HPC = hippocampus; LG = lingual gyrus; IOG = inferior occipital gyrus; SOG = superior occipital gyrus. SUB = subcortex; CBM = cerebellum (reproduced from Groot, Csifcsák, et al. 2021).

Positive correlations with tonic pupil size were observed almost exclusively in subcortical and cerebellar areas, including the thalamus, internal capsule, and intracalcarine cortex of both hemispheres as well as the right hippocampus. In addition, a large brainstem cluster covered the locus coeruleus, substantia nigra, and subthalamic and ventral tegmental nuclei. Furthermore, widespread cerebellar activation was observed in the anterior and posterior lobes and dentate nuclei. Interestingly, brain regions that negatively correlated with tonic pupil size largely overlapped with visual and somatomotor cortical network parcellations, including the primary somatosensory cortices, superior frontal gyri, medial primary motor cortices, lateral occipital cortices, superior temporal gyri, hippocampal areas, and cuneal cortices of both hemispheres in addition to the left middle temporal gyrus and right fusiform gyrus (Fig. 5*A*, Supplementary Material B). In contrast, activity in brain regions that corresponded with changes in phasic pupil responses to task-related events was less and extensive and more posteriorly localized. Specifically, larger phasic pupil responses were associated with greater activity in the left lingual gyrus, whereas smaller phasic pupil responses were associated with activation of the left superior temporal gyrus (poorly visible on the surface mesh), right inferior occipital gyrus, and bilateral superior occipital gyri (Fig. 5*B*).

## Discussion

To disentangle the complex interplay between mind wandering, executive functions, and behavior, we employed an fMRI version of a recently developed finger-tapping random-sequence generation task (FT-RSGT). This novel paradigm allows assessment of ongoing fluctuations in task-related and task-unrelated attentional states through self-reports and sensitive indices of task performance at high temporal resolution. Concurrent fMRI and pupillometric measures were used to investigate the neural substrates of direct and indirect markers of mind wandering.

Participants completed interleaved blocks of two different finger-tapping tasks, one where they performed alternating sequences and one that required randomized responding, that were otherwise identical in terms of stimulus presentation, task pacing, and thought probe frequency. We therefore hypothesized that the difference in neural recruitment between the two tasks should be mainly reflected in the activation of brain regions associated with executive functioning. Indeed, our results demonstrate greater involvement of attention and executive control networks during the generation of random sequences compared to alternating finger-tapping. Specifically, activation was localized in brain regions previously implicated in random number generation, including the right superior parietal lobe and intraparietal sulcus, primary and supplementary motor areas, right dorsolateral prefrontal cortex (DLPFC), anterior insula, and cerebellum (Mattay et al. 1998; Jahanshahi et al. 2000; Gountouna et al. 2010). The DLPFC has been proposed to play an especially important role in the suppression of repetition in response patterns (Jahanshahi et al. 1998; Jahanshahi et al. 2000; Joppich et al. 2004; Capone et al. 2014) and is a major node in the frontoparietal control network (FPCN) that is typically associated with strategic planning and goal-directed cognition. Thus, the coordinated activity of the DLPFC together with somatomotor areas, insula, and cerebellum likely orchestrates the complex behavior required for this task, including the evaluation and selection of spatiotemporal motor actions, suppression of sequence reiterations, and synchronization of responses to an externally-cued rhythm.

In addition, we observed a similar pattern of frontoparietal cortical activation associated with increases in sequence randomness, including the right intraparietal sulcus, right posterior middle frontal gyrus, and left medial supplementary motor area. This is consistent with an early study employing a finger-tapping task combined with random sequence generation (Schubert et al. 1998) and suggests that these regions are especially important for self-determined action planning and execution. In particular, the intraparietal sulcus has been argued to serve important integrative functions of sensorimotor information required to monitor the ongoing movement sequence and adapt new movements according to the randomness criterion (Schubert et al. 1998; Tanabe et al. 2005). However, the absence of correlated activity in the DLPFC with sequence randomness was surprising given previous findings (Jahanshahi et al. 2000; Joppich et al. 2004). Interestingly, a recent study reported a similar dissociation as anodal tDCS of the left DLPFC failed to modulate sequence randomness as measured with approximate entropy (Boayue et al. 2020). Since we did not find evidence for a difference in mind wandering propensity between the two tasks, it is possible that the DLPFC is not necessary for the optimization of sequence entropy but is rather involved in the task-specific coordination and distribution of executive control processes that may also periodically engage in task-unrelated processes such as mind wandering. This interpretation is in line with the proposed role of the DLPFC in context regulation (Turnbull et al. 2019) and conforms to the observation that anodal tDCS of the DLPFC reduced involuntary shifts in task-unrelated attention (Boayue et al. 2020).

Decreases in randomness, as would be expected to occur when thoughts drift away from the task as subjects engage in mind wandering, were instead associated with activity in the left inferior temporal sulcus and left superior and inferior occipital gyri. Although these regions are not typically associated with decrements in task performance, a large-scale meta-analysis (Spreng et al. 2002) reported that the left inferior temporal sulcus is often associated with autobiographical memory retrieval and default mode functions. However, the ordinal regression analysis did not reveal a clear relationship between approximate entropy of the tapping-sequence and mind wandering, suggesting that mind wandering was less detrimental to this aspect of task performance compared to the maintenance of synchronized finger-tapping to the metronome as reflected in the strong relationship with behavioral variability. As we also observed generally higher tapping variability during the random task, participants were possibly more strongly engaged in maintaining task performance by optimizing sequence randomness, leading to the deterioration of tapping rhythmicity.

When executive task demands were low and participants were required to simply produce an alternating finger-tapping sequence, greater activity was observed in the core nodes of the DMN, namely the posterior cingulate cortex/precuneus and left angular gyrus. Given the context-regulation hypothesis, which states that the propensity to mind wander is adaptively adjusted to environmental demands (Smallwood and Andrews-Hanna 2013), it is conceivable that the less effortful alternating finger-tapping task, as opposed to random sequence-generation, was more facilitative of mind wandering as reflected in greater DMN activation (Mason et al. 2007). However, as we failed to observe activation of the DMN directly preceding self-reports of mind wandering, or any difference in mind wandering propensity between the two tasks, an alternative explanation may be that DMN recruitment rather indicated a greater reliance on automated behavior (Shamloo and Helie 2016; Scheibner et al. 2017; Vatansever et al. 2017). This explanation is in line with our observation of correlated activity in the DMN (ventromedial prefrontal cortex and left superior frontal gyrus) when the rhythm of finger-tapping was more synchronized with the metronome, which is in agreement with previous findings characterizing stable or “in-the-zone” behavior (Esterman et al. 2013, 2014; Kucyi et al. 2016; Kucyi et al. 2017; Yamashita et al. 2020). Together, these results suggest that automatic and repetitive behavior that is considered less effortful might be governed by the DMN and fluctuations in behavioral variability may provide a sensitive marker for changes in attention as reflected in neural correlates of attention rather than sensorimotor networks. Furthermore, increases in finger-tapping variability were predictive of self-reported mind wandering episodes throughout the task, highlighting the robustness of this relationship across different tasks and studies (Bastian and Sackur 2013; Hawkins et al. 2019; Boayue et al. 2020) and additionally substantiating behavioral variability as a sensitive marker for departures from task-focused attention.

We did not observe a difference in mind wandering propensity between the two task conditions, which is surprising given previous studies demonstrating an effect of task difficulty on mind wandering (Seli, Konishi, et al. 2018; Brosowsky et al. 2021). One possible explanation as to why levels of mind wandering during the less demanding alternating finger-tapping task were similar to those during the random task is that exposure to the former, easier task was limited. Specifically, alternating blocks composed only one-third of the experiment and were pseudo-randomized, making it unlikely for two alternating blocks to occur sequentially. Therefore, occasional periods of 1-min lasting alternating finger-tapping were possibly not long enough to induce significantly more mind wandering, resulting in comparable levels of mind wandering between the two tasks.

Contrary to expectations, the patterns of neural recruitment directly preceding mind wandering self-reports and during periods of increased tapping variability did not converge. However, the observed divergence in our study is less surprising given the results of a previous study employing a continuous performance task (Kucyi et al. 2016), in which the authors demonstrated greater activation of the DMN in relation to self-reported mind wandering as well as stable performance even though mind wandering was preceded by increases in response variability. Combined with our results, these findings suggest a certain level of independence in the relationships between the DMN and mind wandering on the one hand, and between the DMN and behavioral variability on the other. Although the authors of that study report DMN activation prior to mind wandering, our results failed to show such a relationship. Instead, we observed that mind wandering self-reports were preceded by local activation of the left inferior occipital gyrus and cerebellum during the alternating task and the right striatum during the random task. Regardless of the discrepancy with the neural recruitment during task performance, these results are puzzling on their own as they strongly deviate from the neural regions typically associated with mind wandering and spontaneous thought (Christoff et al. 2009; Fox et al. 2015). However, several studies indicate that cerebellar regions are functionally connected to cortical intrinsic connectivity networks, including the DMN (Habas et al. 2009; Buckner et al. 2011; Vatansever et al. 2015; Habas 2021), revealing a role for the cerebellum in cognition. In addition, earlier findings are suggestive of a role for the striatum in brain state maintenance through connections with the insula in order to sustain mind wandering episodes (Tang et al. 2012; Chou et al. 2017) and a recent study reported the thalamus and basal forebrain as subcortical nodes of the DMN (Alves et al. 2019). Furthermore, a recent study analyzing the dynamics within and between large-scale networks observed that mind wandering interacted with changes in the segregation and integration of visual and subcortical networks (Zuberer et al. 2021). Specifically, mind wandering was associated with higher levels of integration of the visual network compared to optimal sustained attention, whereas the subcortical network showed stronger segregation, suggesting that visual and subcortical system dynamics are sensitive to perturbations from mind wandering. Hence, although the role of cerebellar and subcortical regions and cortico-subcortical network interactions in mind wandering is currently understudied, these findings warrant consideration for future research.

It should be noted that there exists a large heterogeneity in the design and the direct or indirect measurement of mind wandering across previous studies and different forms of mind wandering or spontaneous thought can be discerned based on their neural correlates (Fox et al. 2015). In addition, some researchers have proposed a distinction between stimulus-independent versus stimulus-oriented mind wandering (Gilbert et al. 2007; Maillet et al. 2017), a dimension that cannot be directly investigated in most continuous performance and sustained attention tasks that implement ongoing stimulus delivery. Instead, the FT-RSGT can be considered mostly a stimulus-independent paradigm and thus task performance in general relies more prominently on internal representations. An intriguing speculation arising from these considerations is that network configurations supporting such representations might be similar for task-related and task-unrelated processes, which would explain the absence in neural contrast. Alternatively, the divergence in brain activation identified through direct (self-report) versus indirect (objective) measures may arise from the difference in how they relate to its heterogeneous phenomenological aspects. For example, experience sampling may capture a wide variety of types of spontaneous thought, including episodes that are brief versus prolonged, aware versus unaware, and deliberate versus involuntary. Indeed, there is evidence that the spontaneous generation of mind wandering and its subjective experience are separable components (Smallwood et al. 2007) that can also be distinguished on the neural level (Christoff et al. 2009). In contrast, indices of objective performance may consistently “catch” a distinct and uniform aspect of mind wandering, such as its depth or intensity. As there is evidence that mind wandering without meta-awareness is more disruptive of task performance (Smallwood et al. 2007, 2008), increases in behavioral variability may especially reflect deep and unaware episodes of task disengagement. Future studies are necessary to further investigate these hypotheses.

Finally, we investigated the neural correlates of changes in slowly-fluctuating pupil dilations and constrictions as well as changes in the amplitude of evoked transient responses to task-related events as derivatives of tonic and phasic LC/NE dynamics, respectively (Aston-Jones and Cohen 2005). In agreement with previous reports, spontaneous tonic pupil dilations were correlated with activity in occipito-temporal regions, thalamus, brainstem, and cerebellum, whereas negative correlations were observed within widespread visual and somatomotor cortical areas (Murphy et al. 2014; Yellin et al. 2015; Schneider et al. 2016; DiNuzzo et al. 2019). Especially, the involvement of the LC and thalamus is unsurprising given their known role as drivers of cortical arousal and neural gain that is necessary for optimized task performance (Aston-Jones et al. 1991; Aston-Jones and Cohen 2005; Saper et al. 2005) as well as the proposed role of the thalamus in orchestrating attentional switches between internally versus externally-directed awareness (Wang et al. 2014; Cunningham et al. 2016; Sweeney-Reed et al. 2017) and directing attention to episodic memories (Leszczynski and Staudigl 2016). In addition, the somatosensory cortices have been previously associated with spontaneous thought (Fox et al. 2015) as well as visual imagery and thoughts relating to body-centered information during mind wandering (Delamillieure et al. 2010; Fox et al. 2013). As tonic pupil size was negatively related to self-reported mind wandering, the observed activation of somatosensory cortices in association with tonic pupil constriction could therefore reflect involvement in mind wandering episodes.

Interestingly, occipital activation was frequently either directly or indirectly associated with mind wandering, including when sequence randomness decreased (left superior and inferior occipital gyri), during tonic (lateral occipital cortices) and phasic (right inferior and bilateral superior occipital gyri) pupil constriction, and preceding mind wandering reports in the alternating task (left inferior occipital gyrus), possibly suggesting similar underlying cognitive states. It has been argued that through cortical feedback mechanisms, the occipital cortex may play a role in cognition independent from perceptual input, such as internal visual representations that transpire during mental imagery and mind wandering (Kosslyn et al. 2001; Petro et al. 2016). Together with previous work, these findings provide important insights into how tonic and phasic pupil dynamics may operate as indicators of task-unrelated mental states such as mind wandering (Mittner et al. 2016; Konishi et al. 2017; Unsworth and Robison 2018; Groot, Boayue, et al. 2021). The ordinal regression analysis furthermore revealed a significant interaction between the two pupil components, suggesting that phasic responses only demonstrate a negative relationship with mind wandering when tonic pupil size is increased. This is in line with a recently proposed model of mind wandering based on the adaptive gain theory (Mittner et al. 2016), which poses that two distinct task-unrelated states (“active mind wandering” and “off-focus”) are distinguishable based the level of tonic NE. Specifically, active mind wandering is characterized by similar tonic levels as the on-task state reflecting optimal neural gain and arousal, whereas the off-focus state represents an exploratory mode during which brain networks reconfigure to select relevant behavioral goals. If we assume that participants had generally low levels of arousal and vigilance during the FT-RSGT, the observed high levels of tonic pupil size could possibly reflect optimal levels of tonic NE, during which the amplitude of phasic responses maximally discriminate between active mind wandering (low phasic responses indicate task disengagement and perceptual decoupling) and on-task (high phasic responses reflect task-focused attention).

In summary, we demonstrated that the FT-RSGT relies on the recruitment of attentional and executive control networks, providing evidence for our hypothesis that the generation of random as opposed to alternating finger-tapping sequences requires the use of executive resources. Secondly, we observed positive significant relationships between self-reported episodes of mind wandering and time-on-task as well as behavioral variability, replicating earlier findings and validating the use of this task as an fMRI paradigm (Boayue et al. 2020). Finally, we replicated at least partially the neural correlates of indirect markers of mind wandering and arousal state using sensitive indices of behavioral performance and pupillometric measures as derivatives for LC/NE functioning. In contrast, neither the previously reported cortical networks underlying mind wandering, nor the activation patterns associated with task performance could be observed in the neural contrasts preceding thought probes, suggesting a dissociation between indirect and direct (subjective) measures that may underline the vulnerability of thought probing for disentangling the neural underpinning of this heterogeneous mental state. Together, our results add to the growing body of work to better understand the mechanisms of ongoing fluctuations in attention and how various markers of mind wandering relate to each other at both the behavioral and neural level.

## Supplementary Material

GrootJM_etal_2021_supplementary_material_bhab494

supplementB_bhab494

supplementC_bhab494

## Notes

We would like to thank Steven Miletic and Dr Pierre-Louis Bazin from the Integrative Model-based Cognitive Neuroscience research unit at the University of Amsterdam for their help in the preparations and pilot experiments and for the insightful advice regarding the fMRI analyses conducted in this paper, respectively. *Conflict of Interest*: None declared.
