# Supplementary material for: Catching wandering minds with tapping fingers: neural and behavioral insights into task-unrelated cognition
Source: Cereb Cortex. 2022 Jan 17;32(20):4447–63. doi: 10.1093/cercor/bhab494 (PMC9574234; doi:10.1093/cercor/bhab494)
Supplement: GrootJM_etal_2021_supplementary_material_bhab494 [file grootjm_etal_2021_supplementary_material_bhab494.docx]

**Catching wandering minds with tapping fingers: Neural and behavioral insights into task-unrelated cognition**

Josephine M. Groot^1,2^, Gábor Csifcsák^1^, Sven Wientjes^3^, Birte U. Forstmann^2^, Matthias Mittner^1^

^1^ Department of Psychology, UiT – The Arctic University of Norway, Tromsø, Norway

^2^ Integrative Model-based Cognitive Neuroscience research unit, University of Amsterdam, Amsterdam, The Netherlands

^3^ Department of Experimental Psychology, University of Ghent, Ghent, Belgium

**Supplementary Material**

**Supplement A: Approximate Entropy**

Approximate entropy (AE) is a metric developed by Pincus and Kalman (1997) that “*measures the logarithmic frequency with which blocks of length m that are close together remain close together for blocks augmented by one position.*” In other words, AE summarizes the repeatability of specific patterns in a timeseries. The AE of sequence *U* (with length *N*) for 1≤*m*≤*N* is calculated as follows. First, define blocks of length *m* and calculate the distance between two blocks *x*(i) = (*U*(i),*U*(i+1),…,*U*(i+*m*-1)) and *x*(j) = (*U*(j),*U*(j+1),…,*U*(j+*m*-1)) as *d =* max_k_(|*U*(i+*k*-1) – *U*(j+*k*-1)|) for *k*=1,..,*m*, resulting in a matrix of *N*-*m*+1 by *N*-*m*+1. Because we use sequences of zeros and ones (left=0, right=1), this quantity is exactly zero in case the two sub-sequences are identical and one in case at least one element is different. We count the number of times *d* equals 0, and divide by *N*-*m*+1 to get the similarity criterion (*C_i_^m^*) which is a list of length *N*-*m*+1. Finally, we define Ф*^m^* as the average of the logarithms of *C_i_^m^* (over i) and AE, where larger values of AE indicate a greater degree of irregularity, or randomness.

1. $C_{i}^{m}=\frac{1}{N-m+1}\sum_{i=1}^{N-m+1} d_{i}, d_{i}=\left\{ \begin{aligned} 1, \mathrm{if}\max_{k=1, .. ,m} (\left| U\left( i+k-1 \right)-U\left( j+k-1 \right) \right|)=0 \\ 0, \mathrm{otherwise} \end{aligned} \right.$
2. $Ф^{m}=\frac{1}{N-m+1}\sum_{i=1}^{N-m+1} \log C_{i}^{m}$
3. $\mathrm{AE}\left( 0,N \right)(U)=- Ф^{1}$, $\mathrm{AE}\left( m,N \right)\left( U \right)=Ф^{m}- Ф^{m+1}$ for *m*≥1

*References*Pincus S, Kalman RE. 1997. Not all (possibly) “random” sequences are created equal. Proc Natl Acad Sci USA. 94:3513-3518.

**Supplement B: Cerebellum activation maps**

**
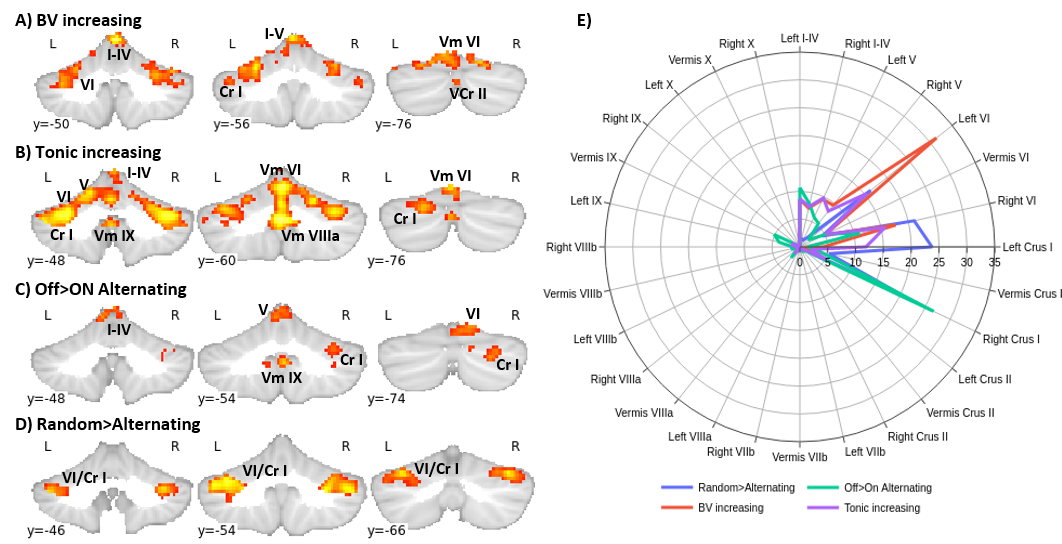
**

**Description.** Coronal views of significant cerebellar cluster activations *A)* correlating with increases in behavioral variability of finger-tapping responses, *B)* correlating with increases in tonic pupil size, *C)* preceding mind wandering reports when contrasted with on-task reports in the alternating task conditions, *D)* during the generation of random sequences when contrasted with alternating finger-tapping, and *E)* the percentage of overlap in every activation map with a cerebellar parcellation (Diedrichsen et al. 2009) in standard MNI152 space. *Vm = vermis; Cr = crus; VCr = vermis crus* (reproduced from Groot et al. 2021b).

**Supplement C: Mind wandering signature across task conditions**

**
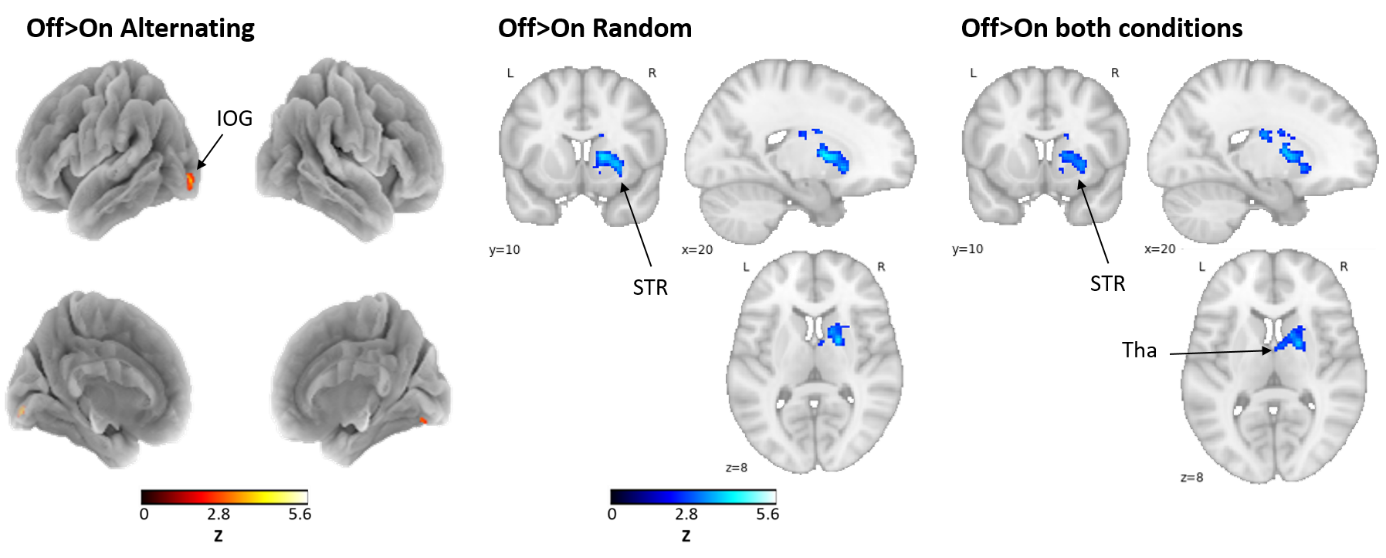
**

**Description**. Active brain regions preceding mind wandering reports when contrasted with on-task reports during the alternating (*left*) and random (*middle*) task conditions separately (reported in the main manuscript), and for all thought probe reports across both task conditions (*right*), showing localized above-threshold activation of the right striatum and right thalamus. The overlap between brain activity preceding mind wandering reports in the random task and across both tasks was .68 (Dice similarity coefficient). *IOG = inferior occipital gyrus; STR = striatum; Tha = thalamus.* (reproduced from Groot et al. 2021b).
